# The BsaHI restriction-modification system: Cloning, sequencing and analysis of conserved motifs

**DOI:** 10.1186/1471-2199-9-48

**Published:** 2008-05-14

**Authors:** Robert K Neely, Richard J Roberts

**Affiliations:** 1 School of Chemistry, The University of Edinburgh, West Mains Road, Edinburgh EH9 3JJ, UK; 2 New England Biolabs Inc., 240 County Road, Ipswich, Massachusetts, 01938, USA

## Abstract

**Background:**

Restriction and modification enzymes typically recognise short DNA sequences of between two and eight bases in length. Understanding the mechanism of this recognition represents a significant challenge that we begin to address for the BsaHI restriction-modification system, which recognises the six base sequence GRCGYC.

**Results:**

The DNA sequences of the genes for the BsaHI methyltransferase, bsaHIM, and restriction endonuclease, bsaHIR, have been determined (GenBank accession #EU386360), cloned and expressed in *E. coli*. Both the restriction endonuclease and methyltransferase enzymes share significant similarity with a group of 6 other enzymes comprising the restriction-modification systems HgiDI and HgiGI and the putative HindVP, NlaCORFDP, NpuORFC228P and SplZORFNP restriction-modification systems. A sequence alignment of these homologues shows that their amino acid sequences are largely conserved and highlights several motifs of interest. We target one such conserved motif, reading SPERRFD, at the C-terminal end of the bsaHIR gene. A mutational analysis of these amino acids indicates that the motif is crucial for enzymatic activity. Sequence alignment of the methyltransferase gene reveals a short motif within the target recognition domain that is conserved among enzymes recognising the same sequences. Thus, this motif may be used as a diagnostic tool to define the recognition sequences of the cytosine C5 methyltransferases.

**Conclusion:**

We have cloned and sequenced the BsaHI restriction and modification enzymes. We have identified a region of the R. BsaHI enzyme that is crucial for its activity. Analysis of the amino acid sequence of the BsaHI methyltransferase enzyme led us to propose two new motifs that can be used in the diagnosis of the recognition sequence of the cytosine C5-methyltransferases.

## Background

DNA restriction-modification (R-M) systems are valuable tools for molecular biology and the methyltransferases in particular, which have well conserved structures [1], also represent excellent model systems for studying the specific interactions between DNA and DNA-binding enzymes. Despite the large number of cloned and sequenced R-M systems [2], in comparison to unique recognition sequences, there is remarkably little sequence similarity amongst the restriction enzymes and, though to a lesser extent, between the target recognition domains of the methyltransferases implying a diverse ensemble of DNA recognition modes and methods is used by these enzymes.

We report the cloning, sequencing and subsequent expression and purification of the BsaHI R-M system from *Bacillus stearothermophilus*. These enzymes target the degenerate sequence GRCGYC, where R= G/A and Y = T/C (Chen, W., Pan, X. and Chen, Z. unpublished data. See REBASE [2]). The inherent degeneracy of the DNA recognition by these enzymes provides an opportunity to study directly the mechanism of specific DNA recognition and to examine the question of how this breaks down into degenerate DNA recognition. Furthermore, such enzymes are exciting targets in the ongoing effort to manipulate the recognition sequences of enzymes, particularly for the restriction enzymes [3].

R. BsaHI belongs to the Type II subfamily of restriction enzymes [4]. It recognises a palindromic sequence of bases and cleaves within this sequence between the purine and cytosine bases: GR/CGYC, where '/' is the cutting site. The restriction enzymes are also sub-classified as belonging to one of several 'superfamilies', named for their conserved motifs. Examples of such superfamilies include the PD-(D/E)xK, HNH or GIY-YIG superfamilies [5]. In the present work, we utilise a bioinformatics approach to classify and identify the conserved, putative catalytic motifs of the R. BsaHI enzyme. Subsequent *in vitro *transcription/translation of a series of mutants is used to identify a motif that is crucial for enzymatic activity.

The target recognition domain (TRD) of the methyltransferase enzymes is the least conserved region of the enzymes of this type. However, some amino acids of the TRD are conserved and a previous report [6] has revealed a consensus motif towards the C-terminal end of the TRD that reads (YFW)X(RK)X_5_P(STCA)PT(ILV)(TASV)X_5–16_H(PFYWL). Structural studies have shown that the residues within and around this motif form critical interactions with the DNA duplex [7,8]. This so-called 'TL' motif lies between 10 and 50 amino acids from the conserved methyltransferase motif IX. Trautner *et al *were the first to note that the TL motif was conserved and applied this knowledge to carefully define and modify the target recognition of multi-specific methyltransferase enzymes in domain swapping experiments [9-11]. A key feature of this work was that it showed that the residues lying to the N-terminal side of the TL motif are responsible for the recognition of the base to the 5'- side of the target base for methylation. Later bioinformatic analysis by Cheng and Blumenthal [12] noted that the recognition of the base directly 5'- of the target base for methylation could be correlated to a conserved R or Q/N upstream of the TL motif for recognition of G or C at this position, respectively. We have built on this previous work with a new bioinformatics analysis, in which we show that, to some extent, prediction of the target specificity of a given methyltransferase is possible by examination of the residues around the conserved TL motif in the variable region of the enzyme.

## Results and Discussion

**Figure 1:**
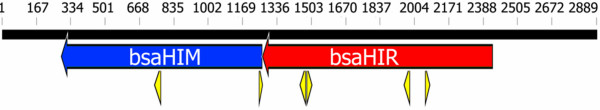
**bsaHIM and bsaHIR**. Schematic showing the relative orientation bsaHIM and bsaHIR genes. The locations of primers used for genome walking are shown as yellow arrows.

Figure 1 shows the relative orientation of the bsaHIM and bsaHIR genes, which are 969 and 1110 bases in length, respectively (GenBank accession #EU386360). The amino acid sequence of the restriction enzyme is in complete agreement with the previously determined N-terminal sequence of this enzyme (J. Benner, unpublished work). The cloned methyltransferase gene lays just six bases upstream of that coding for the restriction enzyme and is initiated with an unusual TTG start codon. An initially expressed enzyme, using the ATG start codon 42 bases from the end of the restriction gene, was found to be inactive. Sequence alignments of the expressed enzyme with other cytosine C5 methyltransferases revealed the absence of the highly conserved Motif I [13] from this enzyme. Extension of the clone to the TTG codon to include the Motif I residues (F [AS]G) in the expressed enzyme recovered the enzymatic activity.

### R. BsaHI

**Figure 2:**
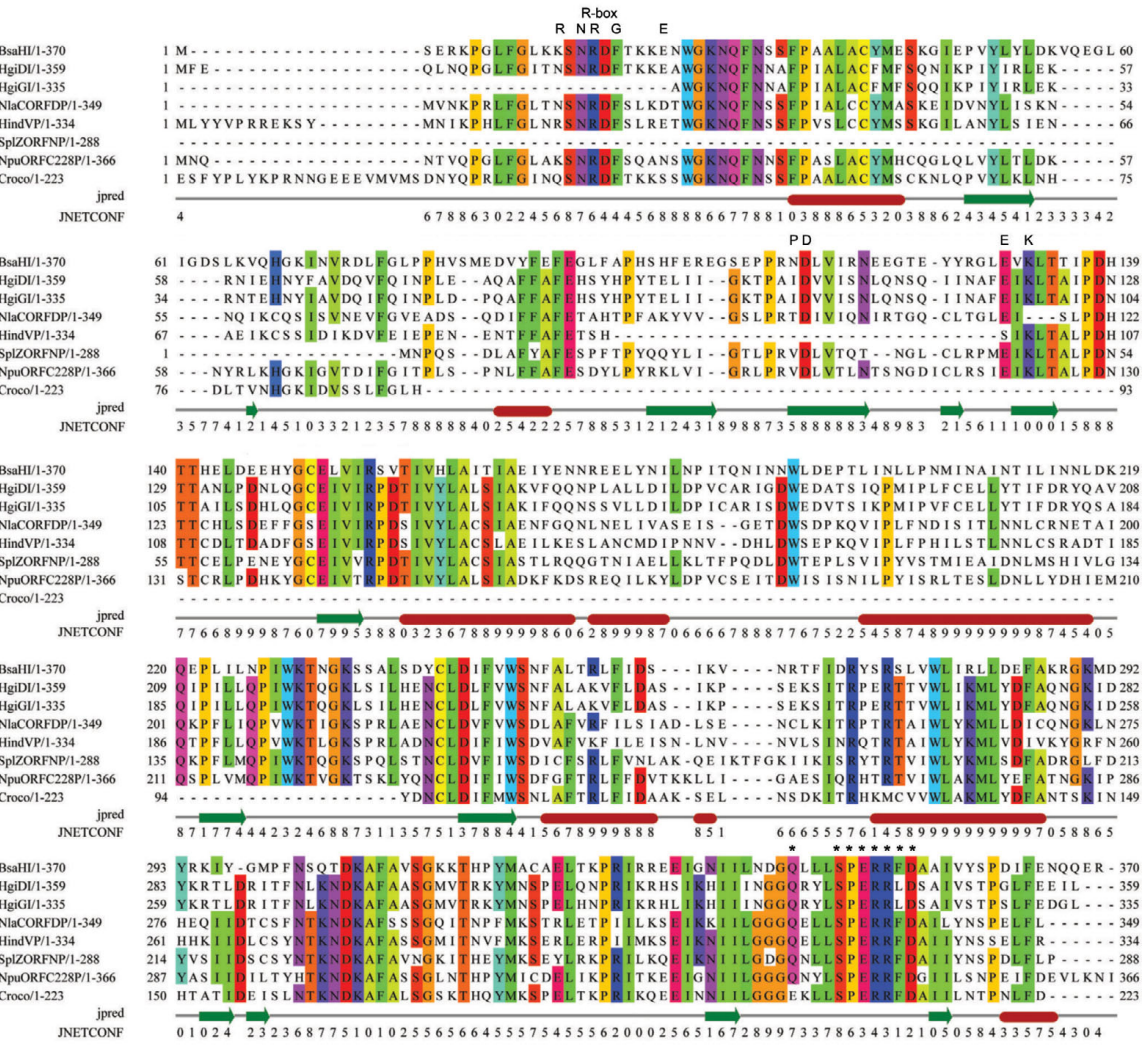
**R. BsaHI Sequence Alignment**. MAFFT alignment of R. BsaHI and its homologues. Residues are coloured where the aligned sequences share at least 60% identity. Proposed conserved and catalytic motifs from the PD-(D/E)xK superfamily are indicated. Also shown is the JNet secondary structure prediction, where green arrows indicate β-sheets and red tubes are predicted α-helices. The JNet prediction confidence parameter, JNETCONF, is shown below the structure prediction. Residues targeted for mutational analysis are indicated with *. Figure generated using Jalview [22].

The amino acid sequence of the R. BsaHI enzyme is strikingly similar to the restriction enzymes belonging to two soil gliding *H. giganteus *bacteria, R. HgiDI and R. HgiGI, which share the BsaHI recognition sequence, GRCGYC. R. BsaHI also shares significant sequence similarity with several putative restriction enzymes: SplZORFNP from *Spirulina platensis*, NlaCORFDP from *Neisseria lactamica*, NpuORFC228P from *Nostoc punctiforme *and HindVP from *Haemophilus influenzae *Rd. Figure 2 shows a MAFFT [14] alignment of these amino acid sequences along with a putative enzyme from the *Crocosphaera watsonii WH85001 *draft sequence, CwatDRAFT_6135.

**Figure 3:**
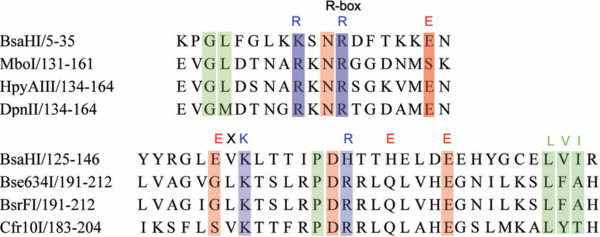
**Alignment of Conserved Motifs Found in the PD-(D/E)xK Superfamily**. Multiple sequence alignments of R. BsaHI with restriction enzymes known to belong to the PD-(D/E)xK superfamily. The 'R-box' motif and region from the proposed catalytic ExK motif of R. BsaHI to the conserved hydrophobic β-sheet (L(153)VI), that forms part of the structural core of the enzyme, are shown.

This group of R. BsaHI homologues show strong conservation of several short amino acid sequences, particularly in the N and C-termini. The central region of the protein has a predicted secondary structure that is consistent with the conserved catalytic core of the PD-(D/E)xK superfamily of restriction enzymes, i.e. a 5-stranded β-sheet, flanked by α-helices. The conserved residues from this family, including the possible catalytic E(130)VK(132) motif (for R. BsaHI), are highlighted in Figure 2. Figure 3 shows a sequence alignment of the putative R-box and ExK motifs with those from endonucleases where these motifs have been established [15,16]. The alignment shows good correlation between the R-box R. BsaHI residues and those of the GATC-recognising enzymes (MboI, HpyAIII and DpnII). These amino acids are important for DNA binding and cleavage in MboI and are likely to be similarly important to the activity of R. BsaHI. Likewise, the ExK and following RxxExxxE motif and conserved hydrophobic β-sheet align well to those motifs in the enzymes recognising RCCGGY (Cfr10I, Bse634I and BsrFI). This is consistent with the putative assignment of EVK(132) as catalytic in R. BsaHI. Notably, the known restriction enzymes (BsaHI, HgiDI and HgiGI) share conservation of this 'ExK' motif but it is absent in three of the five putative enzymes. Thus, we hypothesised that these enzymes should not be active endonucleases. Indeed expression of the HindVP and Crocosphaera genes by *in vitro *transcription/translation revealed that these enzymes have no activity on λ-DNA (unpublished data), consistent with the absence of the 'ExK' motif in these genes and the proposed assignment of these residues as catalytic.

The ExPASy ScanProsite tool was used to carry out a search for enzymes matching any one of three strongly conserved sequence motifs ('WGKNQF', '(Q/K)(T/N)DKAF(A/S)' and 'SPERRFD') from the BsaHI homologues [17]. These motifs were not found beyond the enzymes shown in Figure 2, suggesting that their functionality is specific to these homologues.

**Figure 4:**
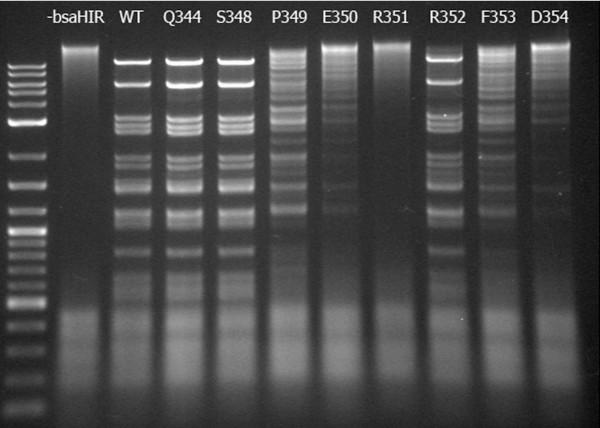
**Activity of R. BsaHI Mutants**. Agarose gel electrophoresis of λ-DNA digested with R. BsaHI mutants. Lane 1 contains the NEB 2-log DNA marker. Control digests were run using the Puresystem without bsaHIR gene (lane -bsaHIR) and with the wild-type bsaHIR gene (lane WT). The remaining lanes are labelled by the amino acid that was mutated to alanine. In all lanes of the gel shown in Figure 4, there is some RNA impurity, resulting from the IVTT mixture. This is seen as a series of diffuse bands running at less than 500 base pairs in length.

Here, we focus on the 'SPERRFD' motif that is conserved at the C-terminal end of the amino acid sequences of the BsaHI homologues. These conserved amino acids are largely capable of forming specific hydrogen bonding interactions and as such could potentially be critical for the enzymatic activity, either as part of the DNA recognition machinery of the enzyme or as part of another intermolecular process, such as dimerisation. We carried out a mutational study in which each of the conserved amino acids in R. BsaHI, Q344 and S348-D354, were mutated to alanine, effectively removing the ability of these residues to form hydrogen bonds or act as bulky, sterically important residues. The mutants were expressed using *in vitro *transcription/translation and the resultant enzymes were incubated with λ-DNA, the digested products of which were separated by electrophoresis on an agarose gel, shown in Figure 4.

Lane "-basHIR" in Figure 4 shows that, in the absence of the bsaHIR gene no sequence-specific digestion takes place. However, a small amount of smearing is evident, indicating that there is a little non-specific nuclease activity in the IVTT mixture. The positive control, with wild-type BsaHI (lane 'WT'), shows complete digestion of the λ-DNA during the four-hour incubation. The Q344A, S348A and R352A mutants all show similar activity and only a small fraction of the DNA is not completely digested. The activity of all of the other mutants has been significantly impaired by the mutation and can be described by P349A~F353A > E350A~D354A > R351A, where the activity of the R351A mutant is negligible.

The similar activity of the Q344A, S348A and R352A mutants to the wild-type R. BsaHI enzyme indicates that these amino acids do not play a functional role in the enzyme. However, all of the other mutations significantly decrease the rate of the digestion. This implies that Q344 and S348 lie in a region of the enzyme that is tolerant of mutation, perhaps a turn or flexible region of the amino acid chain. Those residues from P349 to D354 define a region of the enzyme that is critical to its function. There are clear differences in the digestion rates with the different mutants. The improved activity of the P349A and F353A mutants as compared to the E350A and D354A mutants perhaps indicates that alanine is able to somewhat compensate for the absence of the bulky P/F residues, whereas it clearly cannot mimic the hydrogen bonding functionality of the E/D residues. Remarkably, the R351A mutant is inactive. This result becomes more striking when one considers that the mutation of the neighbouring residue, R352A displays activity comparable to that of the wild-type enzyme. The marked difference in the activity of these mutants of identical, adjacent residues suggests a critical and tightly defined role for R351 in ensuring the activity of R. BsaHI.

### M. BsaHI

**Figure 5:**
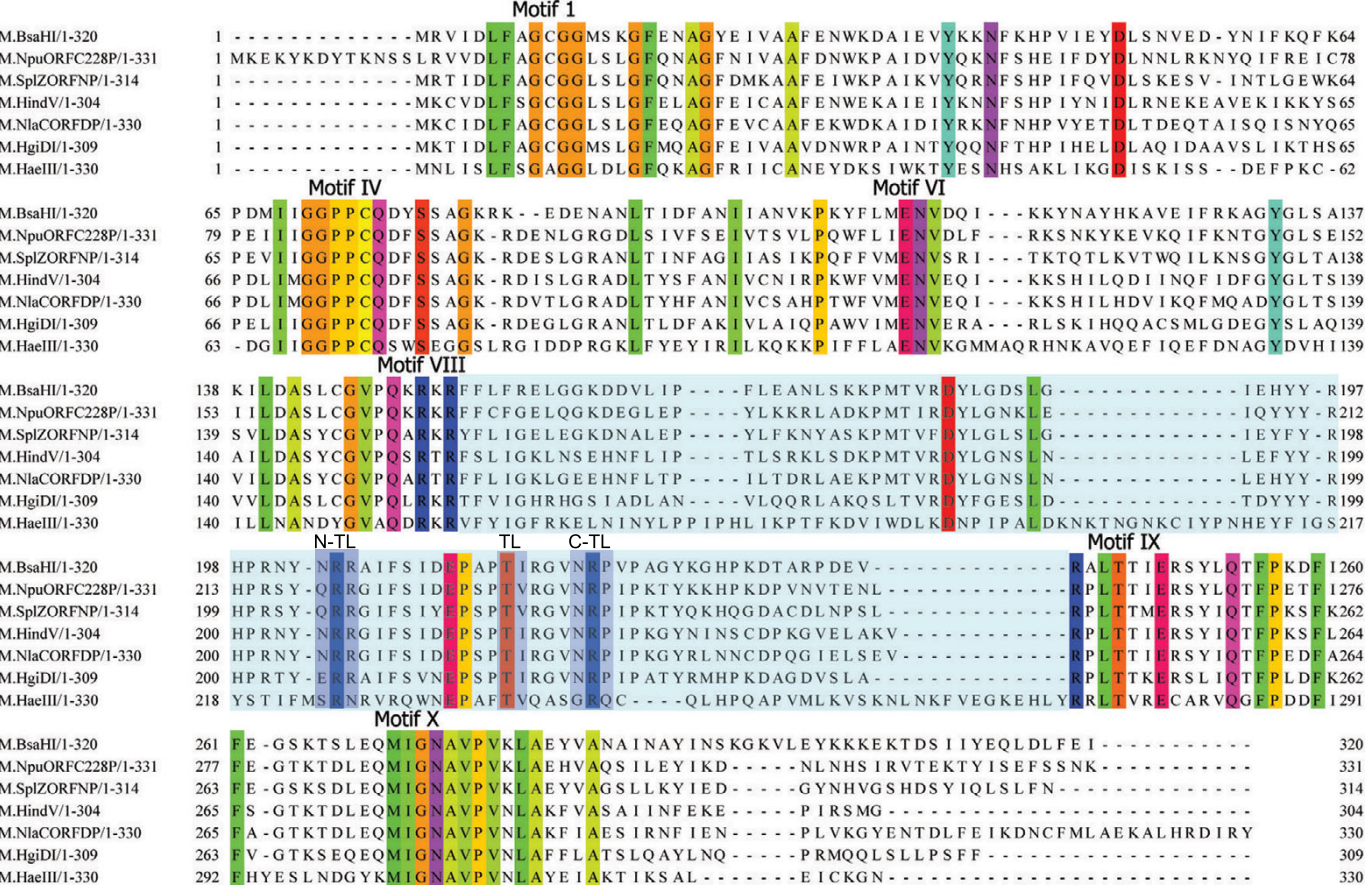
**M. BsaHI Sequence Alignment**. MUSCLE sequence alignment of M. BsaHI and its homologues and M. HaeIII. Residues are coloured where the aligned sequences are completely conserved. Highly conserved motifs are labelled and the highly variable 'target recognition domain' is shaded in light blue. Dark blue shading indicates the proposed N-TL, TL and C-TL motifs.

**Figure 6:**
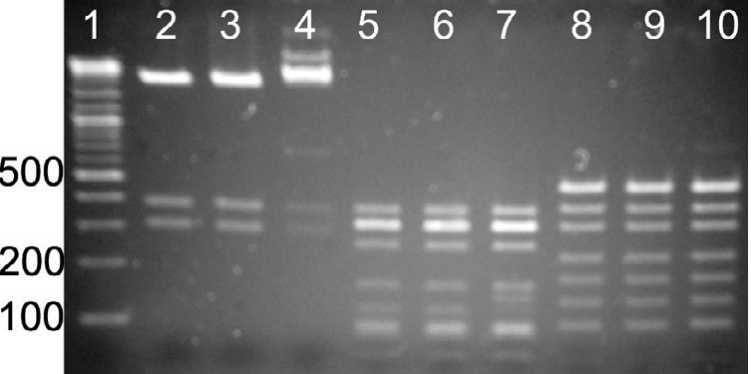
**Determination of Target for Methylation by M. BsaHI**. Agarose gel showing conferred protection against cutting by M. BsaHI. Restriction digests are with R. BsaHI (Lanes 2–4), R. HhaI (Lanes 5–7) and R. HpaII (Lanes 8–10). For each restriction enzyme the three lanes show (from right to left) digest of unprotected DNA, digest of DNA incubated with M. BsaHI but without SAM and the digest of M. BsaHI protected DNA. Note the band that shifts from ~130 bases to ~170 bases in the R. HhaI digests (Lanes 6 and 7), indicating protection against cutting is conferred by M. BsaHI. Figures on the left show the size in bases of the bands in the DNA ladder (NEB 2-log ladder).

Figure 5 shows that the M. BsaHI methyltransferase contains all of the conserved motifs of a cytosine C5 methyltransferase [13]. To determine the target base for methylation, pUC19 plasmid DNA was methylated with the M. BsaHI enzyme. Figure 6 shows the result of subsequent digestion of the DNA with the R. HpaII and R. HhaI restriction enzymes. The single overlapping HhaI/BsaHI site (G**GCGC**C (where boldface bases represent the HhaI recognition sequence and the underlined bases are the BsaHI recognition sequence) was protected from cutting, whereas the overlapping HpaII/BsaHI site (**CC****GG**CGTC) was cut. Since HpaII restriction is blocked by hemi-methylation at the central cytosine of its recognition sequence [2], we conclude that M. BsaHI methylates the central cytosine bases of its GRCGYC recognition sequence. Despite this functional homology to the well-studied M. HhaI, the amino acid sequence of M. BsaHI has little in common with that of M. HhaI beyond the established cytosine C5 methyltransferase structural motifs. Thus, the amino acid sequence of M. BsaHI and its homologues are aligned along with the sequence for M. HaeIII, which also has a known structure [8] but shares more similarity with M. BsaHI, as shown in Figure 5.

**Figure 7:**
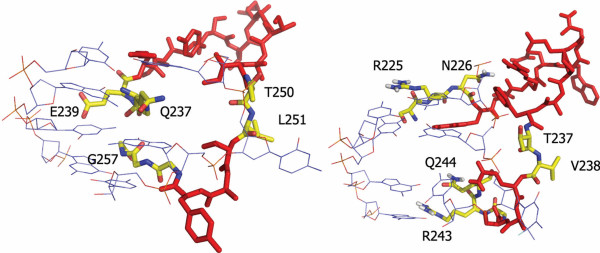
**The TL Motif Structure in M. HhaI and M. HaeIII**. Images taken from crystal structures of M. HhaI (left) and M. HaeIII (right) showing the loop around the conserved TL motif (T250, L251 in M. HhaI and T237, V238 in M. HaeIII). The loop is shown in red with yellow residues indicating the amino acids used in the proposed scheme for describing the methyltransferase recognition sequence. DNA is shown as narrow blue sticks.

The TL motif at the centre of the TRD is shared by M. BsaHI (TI_217_), its homologues and M. HaeIII (TV_238_). The amino acid residues on either side of the TL motif are crucial for DNA recognition [9-11]. For instance, the M. BsaHI homologues share a conserved R with M. HaeIII eleven bases upstream of this motif. In M. HaeIII, this conserved R forms a specific contact to the most 5'-G of the M. HaeIII recognition sequence (Figure 7) and a similar assignment is possible for this residue in M. BsaHI. Cheng and Blumenthal [12] showed that, where the base 5'- of the target cytosine is a guanine, a conserved arginine is often found eight or nine amino acids upstream of the TL motif. In the case of M. BsaHI, nine amino acids upstream from the 'TL', where M. HaeIII is known to be recognising the G directly 5'- to the flipped C, either glycine or alanine is present. The absence of an amino acid capable of forming a specific interaction with the DNA at this position is a possible source of the degeneracy in the M. BsaHI recognition sequence.

Figure 7 shows the superimposed structures of M. HaeIII and M. HhaI and illustrates that the loops on either side of the conserved TL motif are, structurally, well conserved. Using these structures, we define two trimeric sequences on the N-terminal and C-terminal side of the TL motif, which come into close contact with the DNA duplex. These trimers have the spacing 'NNN'x_10_TLx_3_'CCC' and will be referred to as the 'N-TL' and 'C-TL' motifs, henceforth. There is good evidence for the importance of the C-TL motif in the solution phase for M. HhaI [18]. *In vitro *compartmentalisation experiments have shown that G257 is critical to the function of M. HhaI, whereas nearby residues S252 and Y254 can be mutated whilst activity is retained. We hypothesised that, in enzymes using similar mechanisms of DNA recognition and recognising similar sequences, the DNA contacts are likely to be similarly spaced from the TL motif and that these key, DNA-contacting residues are likely to be conserved.

A MUSCLE alignment of the characterised and putative cytosine C5-methyltransferases with known or predicted four base recognition sequences, which contain a clear TL motif, is shown in Additional File 1. For each of the distinct recognition sequences there is conservation of the highlighted N-TL motif and the C-TL motifs. The conservation within these critical regions of the enzymes suggests that, as in M. HhaI and M. HaeIII, these amino acids describe regions involved in DNA recognition and can potentially be employed to diagnose the recognition sequence of the four-base targeting cytosine C5-methyltransferases.

In the case where there is the most sequence information available for characterised enzymes, i.e. those recognising GGCC, the N-TL motif reads exclusively 'SRN'. The C-TL motif is also relatively well conserved with a preference for the trimer 'GRQ'. There are intriguing overlaps in the amino acids used in both the N-TL and C-TL motifs. Most notable are the GCGC recognising enzymes whose C-TL motif reads 'RHG' and the CGCG recognising enzymes, which employ a C-TL motif reading 'HHG'. Similar overlap is seen between the GCGC/CGCG recognising enzymes with N-TL motifs reading 'QGE'/'QG(NQ)' and those recognising CCGG/GGCC with N-TL motifs reading 'ERN'/'SRN' Such overlap is likely an indicator of the common modes of DNA recognition employed by this group of cytosine C5 methyltransferases. The common use of C-TL and N-TL motifs by enzymes recognising opposite recognition sequences (for example GCGC and CGCG) is likely a result of the simple, reversible nature of the hinged structure about the TL motif and implies that this motif is suited to DNA binding in either direction along the duplex.

**Figure 8:**
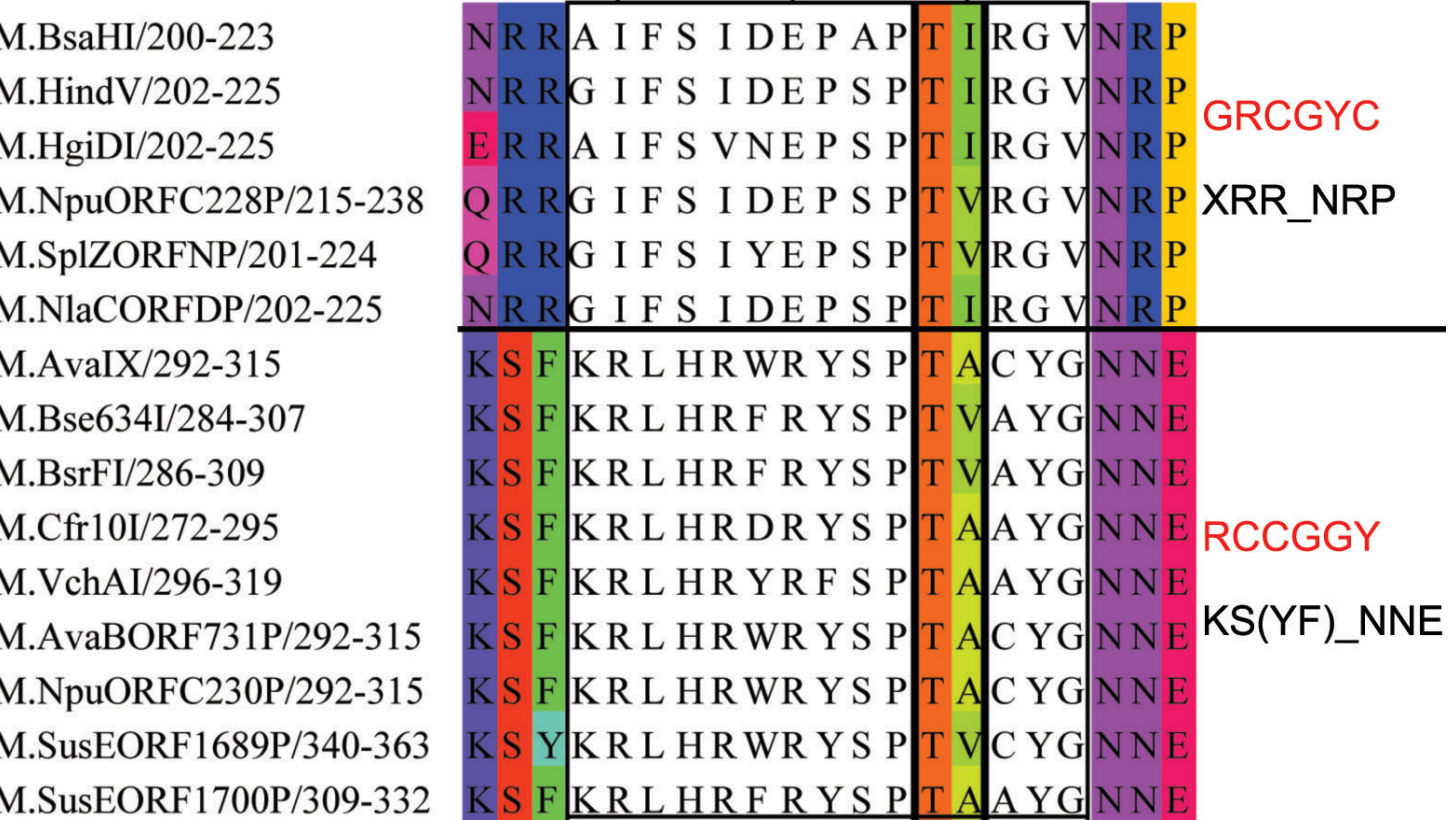
**N-TL and C-TL Motif Alignments for Enzymes Recognising "Five" Bases**. MUSCLE alignment of "five"-base recognising cytosine C5 methyltransferases. Showing the conserved TL motif along with the predicted DNA-recognising amino acids of the N-TL and C-TL motifs. Recognition sequences and those motifs those residues best conserved in the N-TL and C-TL motifs are also shown.

The number of distinct recognition sequences with conserved N-TL and C-TL motifs decreases with increasing length of the target recognition sequence. Of the "five"-base recognising cytosine C5-methyltranferases, there are two, the GRCGYC and YGGCCR recognising enzymes, which have clear TL motifs as shown in Figure 8.

Examination of the amino acid sequences for the six-base recognising enzymes reveals that the cytosine C5 methylating enzymes targeting GTCGAC contain an easily identifiable TL motif. Alignment of the sequences, however, shows that there are no significantly conserved amino acids with the spacing from the 'TL' residues seen for the 4- and 5-base recognising enzymes ('NNN'x_10_TLx_3_'CCC'). Furthermore, although the motif YGRx_8_T(LIM)x_9_GRxGH is well conserved in the GTCGAC recognising enzymes the recently sequenced M. TspMI enzyme, recognising CCCGGG, utilises an almost identical motif (YGRx_8_TIx_9_GRx*L*H). Clearly, the amino acids around the TL motif cannot be used to wholly describe the recognition sequences of the enzymes targeting these relatively long sequences.

## Conclusion

The BsaHI restriction-modification system has been cloned and sequenced. The sequence alignment of R. BsaHI and its homologues clearly shows many highly conserved motifs. We showed through sequence alignment that these enzymes belong to the PD-(D/E)xK superfamily of restriction enzymes and, based on this sequence alignment, have identified residues that are potentially catalytic or involved in DNA binding. We also chose a motif reading 'QxxxSPERRFD' at the C-terminus of R. BsaHI and mutated this to investigate its function. We have shown that this motif is crucial to enzymatic activity and represents a good target for future studies. In particular, we have shown that the R351 residue is critical to the function of R. BsaHI.

M. BsaHI is a cytosine C5 methyltransferase that has been found to methylate the central cytosine of its GRCGYC recognition sequence. The amino acid sequence was found to contain all of the conserved motifs (I to X) for a cytosine C5 methyltransferase. Furthermore, the target recognition domain of the M. BsaHI was found to contain the conserved TL motif. On either side of the TL motif, we identified two amino acid trimers, the N-TL and C-TL motifs, which can potentially be used to diagnose the recognition sequence of the four- and some five-base recognising cytosine C5 methyltransferases. Should these motifs turn out to be reliable indicators of recognition sequence, such information has potential application in the search for restriction enzymes with new specificities, since it should be possible, by simple sequence inspection, to discriminate against genes containing the N-TL and C-TL motifs for known recognition sequences.

## Methods

All enzymes, DNA sequencing reagents and primers were from New England Biolabs Inc. DNA purification was done using spin-column purification (Qiagen) unless otherwise stated. All reagents were used as received and according to the manufacturers instructions.

### Cloning the BsaHI R-M System

The chromosomal DNA encoding the BsaHI R-M system was isolated by phenol extraction from the thermophilic bacterium *Bacillus stearothermophilus*, strain CPW11, from the NEB strain collection. This DNA was partially digested with HpyCH4IV to give an average fragment size of 1–3 kB. Fragments were cloned into the AccI site of pUC19 and subsequently transformed into the methyl-restriction deficient *E. coli *strain ER2566 (NEB T7-Express) using the heat-shock method. The methylase selection method (Hungarian Trick) [19] was used to select clones containing a viable bsaHIM gene. Following two rounds of selection, the isolated clone containing the methyltransferase gene was sequenced. A chromosome walking technique [20,21] was employed in order to sequence the DNA adjacent to the bsaHIM gene. The DNA sequence encoding the bsaHIR gene was located after 3 rounds of inverse PCR, upstream of the methyltransferase gene, as illustrated in Figure 1.

### Sequence alignments

Alignments of the amino acid sequences of the BsaHI R-M and their homologues were carried out using the Jalview sequence alignment editor [22] and generated using the MUSCLE [23] or MAFFT [14] computer programs. Homologues were identified by running a BLAST search, using an E-value cut-off of 1, of the bsaHIR and bsaHIM genes against the restriction/modification enzyme database, REBASE [2].

### Mutations and *In vitro *Transcription and Translation of R. BsaHI

Targeted mutations of R. BsaHI were made using two rounds of PCR. In the first round, fragments of the bsaHIR gene were made using overlapping primers containing the mutated sequences. These fragments were purified and used as complementary primers for the second round of PCR during which a T7 promoter sequence was appended to the 5'-end of the gene. The assembled genes enabled the production of small amounts of wild-type and mutated R. BsaHI protein using the *in vitro *transcription/translation (IVTT) 'Puresystem' from the Post-Genome Institute, Japan. The IVTT system was used according to the manufacturer's instructions. Incubation for 2 h at 37°C resulted in an enzyme concentration equivalent to approximately 0.5 units of the wild-type R. BsaHI per μL (where 1 unit is sufficient to digest 1 μg of λ-DNA in 1 hour). We expect little variation in the expression levels of the mutants of R. BsaHI, although this has not been tested explicitly.

### DNA Cleavage Assay

2 μL of the IVTT mixture was incubated with 500 ng of λ-DNA for 4 h at 37°C in the presence of RNase A. The digested DNA was purified and analysed by electrophoresis on a 1% agarose gel.

### Overexpression and Purification of M. BsaHI

A PCR reaction was carried out to amplify the bsaHIM gene and to append a hexahistidine tag to the C-terminal-end of the gene. The his-tagged gene was cloned into the NheI/EcoRI sites of the pTXBI vector (NEB). This clone was transformed into *E. coli *ER2566, which was grown in Luria Broth in the presence of 100 μg/ml ampicillin at 37°C for 4.5 hrs. Expression of M. BsaHI was induced by addition of isopropyl-β-D-thiogalactopyranoside (IPTG) followed by outgrowth at 30°C for 16 h. The resultant cells (~1 g in 100 ml growth medium) were spun-down and resuspended in 1 ml lysis buffer (50 mM NaH_2_PO_4_, 300 mM NaCl, 10 mM Imidazole, pH 8.0) then subjected to three 20s intervals of sonication. Following centrifugation, the cell extract (~1 ml) was loaded onto a column containing 200 μL Ni-NTA Agarose beads (Qiagen). The his-tagged M. BsaHI was purified from the beads according to the manufacturers instructions. Tests with Bradford's reagent indicated approximately 0.5 mg/ml protein concentration in the second and third (250 μL) elutions from the column.

### Determining the Methylation Target of M. BsaHI

pUC19 plasmid DNA was incubated with M. BsaHI in the presence of SAM for 1.5 h. The methylated DNA (250 ng) was aliquoted to a second reaction containing 0.5 μL of restriction enzyme (R. BsaHI, R. HhaI or R. HpaII) in appropriate buffer. This reaction was incubated for 2 h. The digested DNA fragments were analysed using gel electrophoresis with a 2% agarose gel (Ambion, Agarose-HR) containing 1× SybrSafe dye (Invitrogen).

## Authors' contributions

RKN carried out all experimental work and drafted and edited the manuscript. RJR coordinated the study and revised and edited the manuscript.

## Supplementary Material

Additional file 1 **Image in .jpg format showing N-TL and C-TL motif alignments for enzymes recognising four bases**. Assembled MUSCLE alignments for enzymes with different recognition sequences showing the conserved TL motif along with the predicted DNA-recognising amino acids of the N-TL and C-TL motifs. Amino acids defining particular recognition sequences are shown alongside the alignment, where the residues shown are those best conserved in the N-TL and C-TL motifs. Putative enzymes were disregarded where they did not contain one or more of the recognised methyltransferase motifs IV, VI or VIII or a discrenible TL motif. Click here for file
