# Supplementary material for: The BsaHI restriction-modification system: Cloning, sequencing and analysis of conserved motifs
Source: BMC Mol Biol. 2008 May 14;9:48. doi: 10.1186/1471-2199-9-48 (PMC2413257; doi:10.1186/1471-2199-9-48)
Supplement: Additional file 1 — Image in .jpg format showing N-TL and C-TL motif alignments for enzymes recognising four bases. Assembled MUSCLE alignments for enzymes with different recognition sequences showing the conserved TL motif along with the predicted DNA-recognising amino acids of the N-TL and C-TL motifs. Amino acids defining particular recognition sequences are shown alongside the alignment, where the residues shown are those best conserved in the N-TL and C-TL motifs. Putative enzymes were disregarded where they did not contain one or more of the recognised methyltransferase motifs IV, VI or VIII or a discrenible TL motif. [file 1471-2199-9-48-S1.pdf]

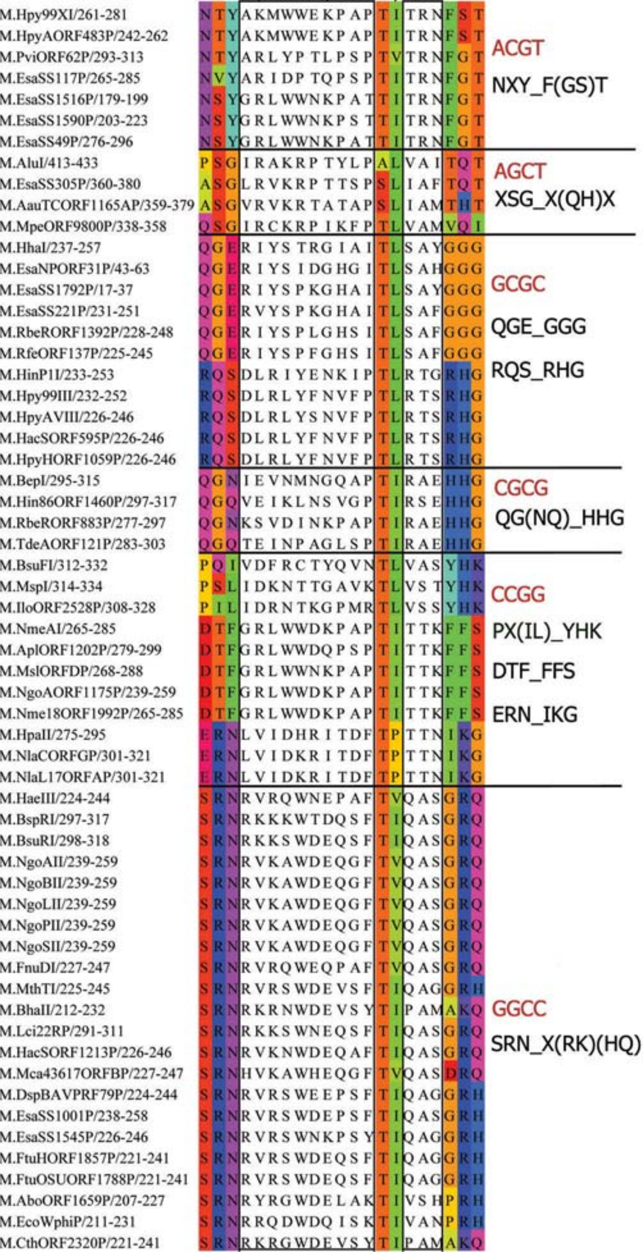
M.Hpy99XI/261-281 NTYAKMWWEKPAPTITRNFST
M.HpyAORF483P/242-262 NTYAKMWWEKPAPTITRNFST
M.PviORF62P/293-313 NTYARLYPTLPSPTVTRNFGT
M.EsaSS117P/265-285 NVYARIDPTQPSPTITRNFGT
M.EsaSS1516P/179-199 NSYGRLWWNKPATTITRNFGT
M.EsaSS1590P/203-223 NSYGRLWWNKPSTTITRNFGT
M.EsaSS49P/276-296 NSYGRLWWNKPATTITRNFGT
ACGT
NXY_F(GS)T
M.AluI/413-433 PSGIRAKRPTYLPALVAITQT
M.EsaSS305P/360-380 ASGLRVKRPTTSPSLIAFTQT
M.AauTCORF1165AP/359-379 ASGVRVKRTATAPSLIAMTHT
M.MpeORF9800P/338-358 QSGIRCKRPIKFPTLVAMVQI
AGCT
XSG_X(QH)X
M.HhaI/237-257 QGERIYSTRGIAITLSAYGGG
M.EsaNPORF31P/43-63 QGERIYSIDGHGITLSAHGGG
M.EsaSS1792P/17-37 QGERIYSPKGHAITLSAYGGG
M.EsaSS221P/231-251 QGERVYSPKGHAITLSAFGGG
M.RbeRORF1392P/228-248 QGERIYSPLGHSITLSAFGGG
M.RfeORF137P/225-245 QGERIYSPFGHSITLSAFGGG
M.HinP1I/233-253 RQSDLRIYENKIPTLRTGRHG
M.Hpy99III/232-252 RQSDLRLYFNVFPTLRTSRHG
M.HpyAVIII/226-246 RQSDLRLYSNVFPTLRTSRHG
M.HacSORF595P/226-246 RQSDLRLYFNVFPTLRTSRHG
M.HpyHORF1059P/226-246 RQSDLRLYFNVFPTLRTSRHG
GCGC
QGE_GGG
RQS_RHG
M.BepI/295-315 QGNIEVNMNGQAPTIRAEHHG
M.Hin86ORF1460P/297-317 QGQVEIKLNSVGPTIRSEHHG
M.RbeRORF883P/277-297 QGNKSVDINKPAPTIRAEHHG
M.TdeAORF121P/283-303 QGQTEINPAGLSPTIRAEHHG
CGCG
QG(NQ)_HHG
M.BsuFI/312-332 PQIVDFRCTYQVNTLVASYHK
M.MspI/314-334 PSLIDKNTTGAVKTLVSTYHK
M.IloORF2528P/308-328 PILIDRNTKGPMRTLVSSYHK
M.NmeAI/265-285 DTFGRLWWDKPAPTITTKFFS
M.AplORF1202P/279-299 DTFGRLWWDQPSPTITTKFFS
M.MslORFDP/268-288 DTFGRLWWNKPAPTITTKFFS
M.NgoAORF1175P/239-259 DTFGRLWWDKPAPTITTKFFS
M.Nme18ORF1992P/265-285 DTFGRLWWDKPAPTITTKFFS
M.HpaII/275-295 ERNLVIDHRITDFTPTTNIKG
M.NlaCORFGP/301-321 ERNLVIDKRITDFTPTTNIKG
M.NlaL17ORFAP/301-321 ERNLVIDKRITDFTPTTNIKG
CCGG
PX(IL)_YHK
DTF_FFS
ERN_IKG
M.HaeIII/224-244 SRNRVRQWNEPAFTVQASGRQ
M.BspRI/297-317 SRNRKKKWTDQSFTIQASGRQ
M.BsuRI/298-318 SRNRKKSWDEQSFTIQASGRQ
M.NgoAII/239-259 SRNRVKAWDEQGFTVQASGRQ
M.NgoBII/239-259 SRNRVKAWDEQGFTVQASGRQ
M.NgoLII/239-259 SRNRVKAWDEQGFTVQASGRQ
M.NgoPII/239-259 SRNRVKAWDEQGFTVQASGRQ
M.NgoSII/239-259 SRNRVKAWDEQGFTVQASGRQ
M.FnuDI/227-247 SRNRVRQWEQPAFTVQASGRQ
M.MthTI/225-245 SRNRVRSWDEVSFTIQAGGRH
M.BhaII/212-232 SRNRKRNWDEVSYTIPAMAKQ
M.Lci22RP/291-311 SRNRKKSWNEQSFTIQASGRQ
M.HacSORF1213P/226-246 SRNRVKNWDEQAFTIQASGRQ
M.Mca43617ORFBP/227-247 SRNHVKAWHEQGFTVQASDRQ
M.DspBAVPRF79P/224-244 SRNRVRSWEEPSFTIQAGGRH
M.EsaSS1001P/238-258 SRNRVRSWDEPSFTIQASGRH
M.EsaSS1545P/226-246 SRNRVRSWNKPSYTIQAGGRH
M.FtuHORF1857P/221-241 SRNRVRSWDEQSFTIQAGGRH
M.FtuOSUORF1788P/221-241 SRNRVRSWDEQSFTIQAGGRH
M.AboORF1659P/207-227 SRNRYRGWDELAKTIVSHPRH
M.EcoWphiP/211-231 SRNRRQDWDQISKTIVANPRH
M.CthORF2320P/221-241 SRNRKRGWDEVSYTIPAMAKQ
GGCC
SRN_X(RK)(HQ)
